# Supplementary material for: A mouse model of hereditary hemorrhagic telangiectasia generated by transmammary-delivered immunoblocking of BMP9 and BMP10
Source: Sci Rep. 2016 Nov 22;5:37366. doi: 10.1038/srep37366 (PMC5118799; doi:10.1038/srep37366)
Supplement: Supplementary Information [file srep37366-s2.pdf]

Supplementary Material

A mouse model of hereditary hemorrhagic telangiectasia generated by transmammary-delivered immunoblocking of BMP9 and BMP10

Santiago Ruiz[1], Haitian Zhao[1], Pallavi Chandakkar[1], Prodyot K. Chatterjee[2], Julien Papoin[3], Lionel Blanc[3,4], Christine N. Metz[2,4], Fabien Campagne[5,6], Philippe Marambaud[1,*]

[1]Litwin-Zucker Research Center for the Study of Alzheimer's Disease, [2]Center for Immunology and Inflammation, and [3]Center for Autoimmune and Musculoskeletal Disorder, The Feinstein Institute for Medical Research, Manhasset, NY, USA. [4]Hofstra Northwell School of Medicine, Hempstead, NY, USA. [5]The HRH Prince Alwaleed Bin Talal Bin Abdulaziz Alsaud Institute for Computational Biomedicine and [6]Department of Physiology and Biophysics, The Weill Cornell Medical College, New York, NY, USA. Correspondence and requests for materials should be addressed to P.M. (email: PMaramba@northwell.edu).

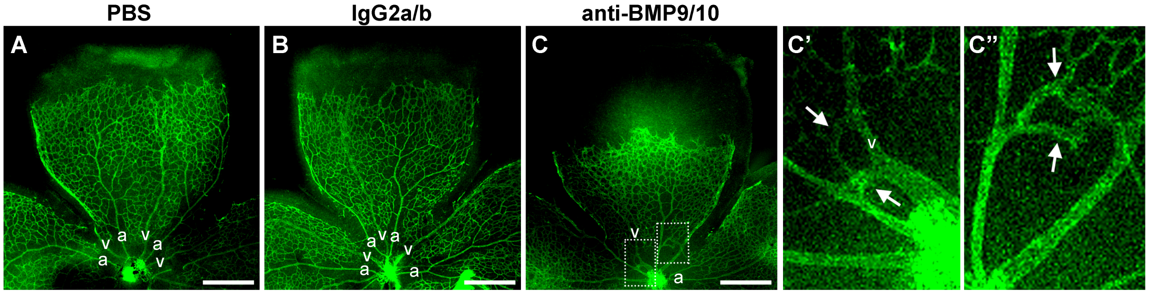

Figure S1: Direct injection of BMP9 and BMP10 blocking Abs leads to abnormal hypervascularization and AVMs in neonatal retinas. (A-C'') Representative images of fluorescent isolectin B4-stained retinas from P6 neonates directly injected with PBS (A), control IgG2a/b Abs (B), or BMP9/10 blocking Abs (C). C' and C" are higher magnification images of the relevant boxed areas in C. a, artery; v, vein; scale bars, 500 μm.

The following document presents the RNA-Seq data analysis conducted for this manuscript in MetaR (http://metaR.campagnelab.org, for a description of the language used to express the analyses, see MetaR: simple, high-level languages for data analysis with the R ecosystem. Fabien Campagne, William ER Digan, Manuele Simi BioRxiv http://dx.doi.org/10.1101/030254).

Gene counts obtained with GobyWeb were analyzed with the following MetaR code:

Figure S2. GobyWeb counts are imported into MetaR. Columns are manually tagged with annotations that indicate what type of data the column contains (see SF2 for definition of the column groups used to annotate columns).

```
Table QKJBKNK-counts-table.tsv  -
File Path
  ${PM_RETINA}/data/QKJBKNK-counts-table.tsv
Columns
  element-id: string [ ID ]
  element-type: [one of: GENE]
  KJLVBLC-PM-R1-Retina-IGg2a2b : numeric [ counts, IgGs ]
  XYYUDDK-PM-R9-Retina-BMP9 : numeric [ counts, BMP9/10 ]
  OPAEERQ-PM-R8-Retina-BMP9 : numeric [ counts, BMP9/10 ]
  IVRLWSH-PM-R2-Retina-IGg2a2b : numeric [ counts, IgGs ]
  KBXIQDP-PM-R5-Retina-IGg2a2b : numeric [ counts, IgGs ]
  OVCXUYC-PM-R6-Retina-IGg2a2b : numeric [ counts, IgGs ]
  UVRMRBK-PM-R12-Retina-BMP9 : numeric [ counts, BMP9/10 ]
  TCUEBZA-PM-R10-Retina-BMP9 : numeric [ counts, BMP9/10 ]
  DIFAXQW-PM-R3-Retina-IGg2a2b : numeric [ counts, IgGs ]
  DXHRWYD-PM-R4-Retina-IGg2a2b : numeric [ counts, IgGs ]
  HKOEODD-PM-R11-Retina-BMP9 : numeric [ counts, BMP9/10 ]
  GSUOHEC-PM-R7-Retina-BMP9 : numeric [ counts, BMP9/10 ]
```

Figure S3. Column Group Definitions. SF1 refers annotated columns with groups. The MetaR column group and usage defines these groups and their relationships (e.g., IgGs and BMP9/10 are two types of Treatment).

```
   Column Groups and Usages

Define Usages:
  Treatment
  heatmap

Define Groups:
  IgGs used for Treatment heatmap
  BMP9/10 used for Treatment heatmap
  Statistics used for << ... >>
  Annotation used for << ... >>
  ignore used for << ... >>
  counts used for << ... >>
  ID used for << ... >>
  GeneName used for << ... >>
  NoGroup used for << ... >>
```

Figure S4. Differential Expression Analysis and Heat Map Construction. This figure presents the main analysis, which call genes differentially expressed with Limma Voom, joins normalized expression values, reduces the results to significantly differentially expressed genes (FDR<=1%) and abs(log2 FC)>=0.7 to reduce the size of the heat map to fit on the figure), adds gene names from Biomart, and constructs a heat map. The multiplot statement offers a preview of the heat map (not shown here, see Figure 3 of the manuscript). Finally, results are written to a tab delimited file.

```
Analysis DiffExp Re tina BMP9
{
  import table QKJBKNK-counts-table.tsv
  limma voom counts= QKJBKNK-counts-table.tsv model: ~ 0 + Treatment
    comparing BMP9/10 - IgGs -> stats: results normalized: normalized
  join ( normalized, results ) by group ID -> joined
  subset rows joined when true: $(adj.P.Val) <= 0.01 & ($(logFC) >= 0.7 | $(logFC) <= -0.7) -> 1% FDR
  transform table 1% FDR -> 1% FDR one ID {
      drop column element-id
  }
  query biomart database Ensembl Genes 83 and dataset Mus musculus genes (GRCm38.p4)
     get attributes Ensembl Gene ID from feature  of types string with column group annotation  ID
                    MGI symbol from feature  of types string with column group annotation  GeneName
     filters Ensembl Gene ID(s) [e.g. ENSG00000139618]  from  1% FDR one ID  <no rowFilter>
     -> geneNames
  join ( 1% FDR one ID, geneNames ) by group ID -> with gene names
  // at this point, with gene names contains 3 columns with ID group. Drop 2 to make it clea  r which one to use for heatmap

  transform table with gene names -> one ID column only {
      drop column genes
      drop column Ensembl_Gene_ID_from_feature
  }
  heatmap with one ID column only select data by one or more group BMP9/10, group IgGs -> heatmap no style [
    show names using group  GeneName
    annotate with these groups:  Treatment
    scale values: scale by row
    cluster columns: true cluster rows: true
  ]
  multiplot -> preview [  1 cols x 1 rows ]  Preview
   [ heatmap ]
  render heatmap as PDF named "heatmap-RETINA-2.pdf "  -  no style
  write with gene names to "selected-RETINA-normalized.tsv "  -
}
```

Figure S5. Annotation of differentially expressed genes. This analysis retrieves gene annotations using BioMart, joins the annotation with the normalized read count table, reorders columns and writes the annotated results to a file.

```
Analysis Annotate selected
{
  import table selected-RETINA-normalized.tsv
  query biomart database Ensembl Genes 83 and dataset Mus musculus genes (GRCm38.p4)
    get attributes Ensembl Gene ID from feature  of types string with column group annotation  ID
                   MGI symbol from feature  of types string with column group annotation  Annotation
                   Description from feature  of types string with column group annotation  Annotation
    filters Ensembl Gene ID(s) [e.g. ENSG00000139618]  from  selected-RETINA-normalized.tsv  when true: $(logFC) != 0
    -> resultFromBioMart
  join ( selected-RETINA-normalized.tsv , resultFromBioMart ) by group ID -> annotated
  reorder columns  in table  annotated  {  UP  DOWN  group ID      -> reordered
                                           UP  DOWN  group Annotation
                                           UP  DOWN  group IgGs
                                           UP  DOWN  group BMP9/10
                                           UP  DOWN  group Statistics
                                         }
  write reordered to "annotated-RETINA-0.7-normalized.tsv "  -
}
```

Table S1 presents the annotated results (annotated-RETINA-0.7-normalized.tsv, imported into Excel) and describes differentially expressed genes with a log 2 fold change larger than 0.7 (in absolute value).
